# Health care workers’ knowledge, attitudes and practices on tuberculosis infection control, Nepal

**DOI:** 10.1186/s12879-017-2828-4

**Published:** 2017-11-17

**Authors:** Anita Shrestha, Dipesh Bhattarai, Barsha Thapa, Prem Basel, Rajendra Raj Wagle

**Affiliations:** 1 University of Queensland, Brisbane, QLD Australia; 2 Queensland University of Technology, Brisbane, QLD Australia; 3 Save the Children in Nepal/Regional TB Center, Kaski, Pokhara, Nepal; 4 Department of Community Medicine and Public Health, Institute of Medicine, Tribhuvan University, Kathmandu, Nepal

**Keywords:** Tuberculosis, Infection control, Health care workers, Knowledge, Attitudes and practices

## Abstract

**Background:**

Infection control remains a key challenge for Tuberculosis (TB) control program with an increased risk of TB transmission among health care workers (HCWs), especially in settings with inadequate TB infection control measures. Poor knowledge among HCWs and inadequate infection control practices may lead to the increased risk of nosocomial TB transmission.

**Methods:**

An institution-based cross-sectional survey was conducted in 28 health facilities providing TB services in the Kathmandu Valley, Nepal. A total of 190 HCWs were assessed for the knowledge, attitudes and practices on TB infection control using a structured questionnaire.

**Results:**

The level of knowledge on TB infection control among almost half (45.8%) of the HCWs was poor, and was much poorer among administration and lower level staff. The knowledge level was significantly associated with educational status, and TB training and/or orientation received. The majority (73.2%) of HCWs had positive attitude towards TB infection control. Sixty-five percent of HCWs were found to be concerned about being infected with TB. Use of respirators among the HCWs was limited and triage of TB suspects was also lacking.

**Conclusions:**

Overall knowledge and practices of HCWs on TB infection control were not satisfactory. Effective infection control measures including regular skill-based training and/or orientation for all categories of HCWs can improve infection control practices in health facilities.

## Background

Tuberculosis (TB) remains a global public health threat affecting millions of people every year [1]. With the increasing incidence of drug resistance and HIV pandemic, TB control efforts have now become even more challenging and this has led to a greater concern towards TB infection control (IC). Institutional settings, including health care facilities, have been identified to be at high risk of TB transmission [2]. Studies have reported nosocomial transmission of TB with high TB infection among health care workers (HCWs) in many countries particularly in low- and middle-income countries [3–7]. This increased risk of TB transmission in health facilities put HCWs and other patients at high risk of infection leading to a serious threat particularly when concerned about drug-resistant (DR) and HIV associated TB [8].

Global experts recommend IC as one of the key strategies for TB control and there are TB IC guidelines available even for the resource-poor settings [9]. However, its implementation seems to be inadequate in many settings. Several studies have reported poor TB IC measures in health facilities [3, 10–12]. Further, HCWs are practicing without adequate IC training and often lack knowledge on TB IC [13, 14]. This is likely to contribute to the increased risk of nosocomial transmission of TB.

Like many other developing countries, TB is a priority public health problem in Nepal. There were an estimated 44,000 incident TB cases (156 per 100,000) and 6100 TB deaths in 2015 [1]. Drug resistance level is high with approximately 9.3% of new cases resistant to at least one drug. The rate of multi-drug resistant TB is 2.2% among new cases while it is 15.4% among retreatment cases [15]. Though HIV prevalence in the country is low, which is estimated at 0.2% among adult population [16], there have been reported high rates of TB/HIV coinfection [17–19]. The latest surveillance (2012) showed that HIV prevalence among TB cases is 2.4% while TB prevalence among HIV clients is 11.5%. High levels of drug resistance and TB/HIV coinfection add more concerns towards IC, which remains a key challenge in Nepal [15]. National TB Programme (NTP) has not made significant effort to address the issue and no focal person has been assigned for IC at the national level. There does not exist any policy and/or guideline on TB IC to assist implementation of IC measures in health facilities. Information on TB IC knowledge and practices among HCWs can provide an important basis for NTP to initiate effort towards TB IC, but the relevant literature is lacking. Therefore, the study assessed knowledge, attitudes and practices (KAP) among HCWs on TB IC.

## Methods

This study formed a part of a broader evaluation of TB infection among HCWs and IC infrastructure assessment in health facilities in three districts of Nepal. An institution-based descriptive cross-sectional survey was carried out in Kathmandu, Lalitpur and Bhaktapur districts. There were 107 Directly Observed Treatment Short-course (DOTS) centres in the area. Due to logistic and time limitation, all health facilities could not be surveyed and therefore, health facilities were randomly selected from the list available from the National Tuberculosis Centre, Nepal. Since the study was a part of TB infection assessment among HCWs, the sample size was calculated based on the proportion of TB infection among HCWs. Due to the lack of information on the prevalence of latent TB infection among HCWs in Nepal, the prevalence was assumed to be 50% as informed by a study in India [20]. The confidence level and margin of error were 95% and 0.6, respectively. All HCWs involved in TB services available during the study period were enrolled until the required sample number was met. Thus, a total of 190 HCWs from 28 health facilities were surveyed. The health facilities included government (17) and non-government (11) facilities providing TB services in the area. Nine of them were hospitals and 19 were primary level health facilities. Since TB service in Nepal is integrated within the national health system and provided through the same outlet often within limited infrastructure, all HCWs are likely to be exposed to TB infection regardless of their job categories. Therefore, the study enrolled different cadres of HCWs who were directly or indirectly involved in TB care, which included physicians, nurses, community health care workers (CHWs) [health assistants, axillary health workers, axillary nurse midwives, and clinic assistants], laboratory and X-ray technicians, administration staff (administration and logistic staff, and TB volunteers), ward attendants and support staff.

A structured questionnaire was developed for data collection which included the specific components of TB IC questions relating to KAP and was based on tools used in similar studies [12, 13]. The tool comprised of HCWs’ demographic and service related variables (age, sex, education, job category, duration of employment, training/orientation on TB and TB IC); knowledge variables (general information on TB and IC measures); attitude variables (statements on TB and IC); and practice variables (time spent with TB suspects or patients, use of respirators, triage and patient education). The tool was translated into the Nepali language, pre-tested and revised as appropriate. Two field workers were trained on the final questionnaire and mobilised for the data collection. They visited the selected health facilities and conducted face-to-face interviews with the HCWs after obtaining informed consent. They were regularly monitored and supervised during the data collection process.

All completed questionnaires were cross-checked and edited on the same day and before data entry to ensure data consistency and completeness. All recorded data were coded and entered in Epi Data. Analyses were performed using Statistical Package for the Social Sciences (SPSS) for Windows software full version 18. Descriptive statistics and bivariate analyses were used for the analysis. HCWs’ knowledge variables were scored and respondents answering ≥60% of the knowledge questions correctly were considered as having good level of knowledge and others were considered to have poor knowledge. The knowledge level was tested for the association with HCWs’ characteristics at significance level of 0.05. The attitude variables comprised of 12 statements with response categories “Agree”, “Disagree” or “Neutral”. Composite scores were calculated and those scoring ≥70% were considered as having positive attitudes towards TB IC. The cut-off points for knowledge and attitude levels were set as informed by similar studies [21, 22].

## Results

Of 190 HCWs, 120 (63.2%) were female and 70 (36.8%) were male. The mean age of HCWs was 36 ± 9.7 (18-59) years. Table 1 shows that 42% of the HCWs were CHWs followed by administration staff (16.3%), ward/support staff (16.3%), nurses (10.5%), lab/X-ray technician (10.5%) and physician (4.2%). Duration of employment of the HCWs ranged from less than 1 year (8.4%) to 10 years and more (37.9%). Nearly one-third of the HCWs (32.2%) reported that they had received some level of training or orientation on TB and most of them were clinical staff (25.3%) followed by lab/X-ray staff (5.3%). Of those who reported receiving TB training and/or orientation, only 12% had received TB IC specific training.

**Table 1:** Sociodemographic characteristics of HCWs

Variables	Categories	Frequency	Percentage
Age	18–30 years	63	33.2
	31–40 years	69	36.3
	Above 40 years	58	30.5
Mean + SD (range)	36 ± 9.7 (18-59)		
Sex	Male	70	36.8
	Female	120	63.2
Educational level	Secondary and below	130	68.4
	Tertiary and above	60	31.6
Job category	Physician	8	4.2
	Nurse	20	10.5
	CHWs	80	42.1
	Lab/X-ray technician	20	10.5
	Ward attendant/support staff	31	16.3
	Administrative staff	31	16.3
Duration of employment	Less than 1 year	16	8.4
	1–4 years	54	28.4
	5–9 years	48	25.3
	10 years and above	72	37.9
Training or orientation on TB	Clinical staff	48	25.3
	Lab/x-ray staff	10	5.3
	Ward attendant/support staff	1	0.5
	Administrative staff	2	1.1
Training or orientation on TB IC	Yes	7	11.5
(*n* = 61)	No	54	88.5

### Knowledge of HCWs on TB IC

Table 2 shows knowledge of HCWs on TB IC. Most of the HCWs were aware of the major symptoms (67.4%) and route of TB transmission (81.6%). However, only half of them (54.7%) could differentiate between TB infection and TB disease. Nine out of ten HCWs stated that use of respirators can prevent TB infection and regarding the IC measures to be implemented in health facilities, 55% of them stated personal respiratory protection followed by environmental controls (47.4%) and administrative controls (14.7%).

**Table 2:** Knowledge of HCWs on TB IC

Knowledge questions on TB IC	Correct response (%)				
	All HCWs (*n* = 190)	Clinical staff (*n* = 108)	X-ray/ Lab staff (*n* = 20)	Admin. staff (*n* = 31)	Ward/ support staff (*n* = 31)
What are symptoms of TB?					
Cough for 2 weeks or more	128 (67.4)	82 (75.2)	12 (63.2)	11 (35.5)	23 (74.2)
Weight loss	143 (75.3)	96(88.1)	14 (73.7)	13 (41.9)	20 (64.5)
Fever	162 (85.3)	101 (92.7)	18 (94.7)	19 (61.3)	24 (77.4)
Loss of appetite	110 (57.9)	69 (63.3)	16 (52.6)	10 (32.3)	13 (41.9)
Chest pain	129 (67.2)	89(81.7)	15 (78.9)	9 (29.0)	16 (51.6)
Blood in sputum	117 (61.6)	75 (68.8)	13 (68.4)	11 (35.5)	18 (58.1)
How is TB transmitted?	155 (81.6)	101(92.7)	16 (84.2)	15 (48.4)	23 (74.2)
What is difference between TB infection and disease?	104 (54.7)	74 (67.9)	13 (68.4)	4 (12.9)	13 (41.9)
Definition of TB suspects	101 (53.2)	66 (60.6)	13 (68.4)	6 (19.4)	16 (51.6)
Which TB is infectious?	155 (81.6)	105 (96.3)	18 (94.7)	12 (38.7)	20 (64.5)
How long is a TB patient infectious after starting treatment?	60 (31.6)	46 (42.2)	5 (26.3)	3 (9.7)	6 (19.4)
In what ways, can HCWs prevent TB infection?					
Use of respirators by HCWs	175 (92.1)	104 (95.4)	16 (84.2)	27 (87.1)	28 (90.3)
Use of masks by patients	65 (34.2)	42 (38.5)	9 (47.4)	3 (9.7)	11 (35.5)
What are IC measures in health facilities?					
Administrative controls	28 (14.7)	16 (14.7)	8 (42.1)	0 (0.0)	4 (12.9)
Environmental controls	90 (47.4)	61 (56)	10 (52.6)	5 (16.1)	14 (45.2)
Personal respiratory protection	105 (55.3)	62 (56.9)	17 (89.5)	10 (32.3)	16 (51.6)

Mean score of knowledge on TB IC among HCWs was 10.56 ± 4.29. The result shows that 54% of HCWs scored “good” level of knowledge while 46% of them had “poor” knowledge level. Table 3 shows that knowledge level of the HCWs was significantly associated with educational status, job category and TB training or orientation received by the HCWs.

**Table 3:** HCWs characteristics and knowledge level on TB IC

HCWs’ characteristics		Knowledge level		*P* value
		Good	Poor	
Age	18–30 years	37 (35.9)	26 (29.9)	
	31–40 years	39 (37.9)	30 (34.5)	0.361
	Above 40 years	27 (26.2)	31 (35.6)	
Sex	Male	41 (39.8)	29 (33.3)	
	Female	62 (60.2)	58 (66.7)	0.357
Educational level	Secondary and below	59 (57.3)	71 (81.6)	0.000*
	Tertiary and above	44 (42.7)	16 (18.4)	
Job category	Clinical staff	72 (69.9)	37 (45.5)	
	Lab/X-ray staff	13 (12.6)	6 (6.9)	0.000*
	Administrative staff	13(12.6)	18 (20.7)	
	Ward/support staff	5 (4.8)	26 (29.9)	
Duration of employment	Less than 5 years	39 (37.9)	31 (35.6)	
	5–9 years	25 (24.3)	23 (26.4)	0.927
	10 years and above	39 (37.9)	33 (37.9)	
Training or orientation on TB	Yes	49 (47.6)	12 (13.8)	
	No	54 (52.4)	75 (86.2)	0.000*

*P value <0.05

### Attitude of HCWs towards TB IC

Our study found that the majority (73.2%) of HCWs had positive attitudes towards TB IC. As shown in Table 4, almost all HCWs agreed that there is a need for a TB IC guideline in health facilities (99.5%). Almost all of them (98.4%) agreed that they should wear respirators while caring for TB patients. Majority of HCWs (64.7%) were concerned about being infected with TB and nearly one fifth of them agreed that cough hygiene alone has no role in TB IC. The analysis shows that HCWs’ attitude level was significantly associated with their level of knowledge on TB IC.

**Table 4:** Attitudes of HCWs towards TB IC

Statement	Agree	Neutral	Disagree
There is a need for guidelines regarding TB IC in a health care facility	189 (99.5)	1 (0.5)	0 (0.0)
HCWs should wear respirators while caring for TB patients	187 (98.4)	1 (0.5)	2 (1.1)
Respirators do not protect against drug-resistant TB even if I wear it all time	95 (50)	7 (3.7)	88 (46.3)
Even after a patient with TB leaves the room I am working in, I remain at risk of contracting TB	163 (85.8)	3 (1.6)	24 (12.6)
Most HCWs are already infected so there is no need of IC measures	12 (6.3)	2 (1.1)	176 (92.6)
I do not wear respirator because patients do not like me to wear it	32 (16.8)	0 (0.0)	158 (83.2)
I am concerned about being infected with TB	123 (64.7)	0 (0.0)	67 (35.3)
There is need to screen HCWs who may be exposed to TB for TB infection or disease	189 (99.5)	0 (0.0)	1 (0.5)
I may turn off fans if they become noisy or cause cold air	155 (81.6)	2 (1.1)	33 (17.4)
Sputum induction procedures in wards put HCWs at an increased risk of getting infected with TB	175 (92.1)	2 (1.1)	13 (6.8)
Cough hygiene has no role to play in IC	36 (18.9)	2 (1.1)	152 (80)
HCWs working in HIV care and treatment clinics are at risk of infection with TB	154 (81.1)	2 (1.1)	34 (17.9)

### Practice of HCWs on TB IC

Table 5 shows that 48% of the HCWs were exposed to TB patients up to 25% of their working day and one-fourth of them usually spent 25 to 50% of their time with TB patients. Thirty-eight percent of them never used respirators and only 10% of them reported using N-95 mask. While enquiring about their practice after seeing a coughing patient in the health facility, most of them (59.5%) replied that they ask the patient to cover his/her mouth while one-fourth (25.3%) of them stated that they do nothing. For patient education, only medical staff were considered as administration and support staff were not supposed to provide health education. Of them, 38% stated that they inform TB patients or suspects of cough etiquette and respiratory hygiene on daily basis.

**Table 5:** Practices of HCWs on TB IC

IC practice	Frequency	Percentage
Proportion of shift spent with TB patients		
Less than 25%	91	47.9
26-50%	47	24.7
51-75%	38	20.0
Above 75%	14	7.4
Proportion of shift worn respirator		
Not at all	73	38.4
Less than 25%	79	41.6
26-50%	18	9.5
51-75%	6	3.1
Above 75%	14	7.4
Type of respirator used		
N-95 mask	19	10.0
Surgical mask	68	35.8
Ordinary mask	30	15.8
What did they do for coughing patient in queue		
Ask duration of their cough	56	29.5
Place them in separate waiting area	6	3.2
Place them in front of queue	6	3.2
Inform about cough etiquette	113	59.5
Do nothing	48	25.3
Patient education on cough etiquette (medical staff only, *n* = 128)		
Always	49	38.3
Sometimes	55	43.0
Never	6	4.7
As per the need	18	14.1

## Discussion

The KAP assessment showed that overall knowledge and practices of HCWs on TB IC were not satisfactory. TB was well understood among HCWs as the majority of them were aware of signs and symptoms of TB, and its transmission. However, TB IC specific knowledge level was poor. Most often HCWs lack proper knowledge in TB IC despite having the good level of understanding on general information regarding TB [13, 23]. The percentage of HCWs scoring “good” knowledge level was 54, relatively less than reported 74% in Ethiopia [21]. The variation in the percentage can be attributed to different methodological approaches where HCWs in the Ethiopian study represented clinical personnel while our study also included administration and other staff.

On contrary to other similar studies [22, 24, 25], we assessed all cadres of HCWs due to their probable exposure to TB infection and having an important role in implementing IC measures in health facilities. Prevention of nosocomial TB transmission requires that all HCWs are aware of TB IC and ensure adequate practices in health facilities. Most often non-medical staff are ignored in training, respirator use, etc. while their risk of being infected and implications in IC interventions cannot be undermined. Not surprisingly, the study revealed significant differences in knowledge level among different categories of HCWs. The knowledge level was relatively good among medical staff compared to administration staff and was much lower among ward/support staff. The higher knowledge level among the medical staff is obvious because they have TB and IC components covered during their study and are also likely to have received relevant training.

Our study has clearly indicated a gap in training and/or orientation on TB IC for HCWs which could have attributed to poor knowledge level particularly among administration and lower qualified staff. NTP Nepal provides regular basic and refresher trainings for various categories of HCWs. However, much of these trainings focus on clinical management of TB and such trainings rarely include non-medical personnel. Furthermore, TB IC specific training or orientation package is lacking, focusing the need of regular training for all HCWs. It is also important to consider how effective are the trainings to bring about desired changes in IC practices. Proper knowledge has been identified to be a predictor of good IC practice and notwithstanding that TB related training is associated with good knowledge, it does not necessarily ensure adequate IC practices [21, 24, 25]. Rather than conventional training, skill-based training utilizing adult learning approaches aimed at modifying HCWs’ behaviour can improve IC practices in the health facilities.

Effective implementation of IC measures in health facilities also relies on HCWs’ appropriate attitudes and motivation. Despite having appropriate attitude towards TB IC, HCWs’ fear for contracting TB is noteworthy. This fear could often be relating to infecting others including family members and the possible financial implications associated after being infected [14, 26]. The study also highlighted HCWs’ realization for the need of regular screening along with the use of respirators while caring for TB patients. However, the use of respirators was very limited. There is no regular supply of respirators especially the N-95 masks for HCWs, which was supported by our observation during IC infrastructure assessment and this probably could be a reason for the limited use. A study [27] has also pointed unavailability of respirators as a barrier to respirators use. Similarly, stigma associated with TB could also be one of the reasons as HCWs reported of avoiding respirators for the comfort of patients. The study also revealed the inadequate practice of triage and poor patient education. Unlike a South African study [13], we noted very low percentage of HCWs providing information on cough etiquette to their patients on daily basis, reflecting the need to focus more on patient education.

The knowledge deficit and inadequate IC practices of HCWs are major barriers to implement IC measures resulting in an increased risk of TB transmission in health facilities. The poor knowledge and practices could be attributed to lack of TB IC policy and/or guideline both at national and institutional levels. NTP Nepal has not yet adapted or formulated the country specific TB IC guideline. Thus, health facilities are not obliged to strictly implement the recommended IC measures and HCWs are not familiar with the IC standards. It is imperative that NTP seriously pay attention towards the issue and this study can provide a sound evidence base to initiate development of TB IC guideline. Given limited resources, NTP may not be able to immediately afford expensive structural and environmental adjustments. As such, cost-effective and sustainable approaches can be taken into consideration. For instance, simply making HCWs aware of necessary IC measures like triage, proper sputum handling, respiratory hygiene and patient education can significantly minimise the risk of TB transmission in the health care settings.

There are certain limitations in our study. The study was conducted in a small population limiting the generalizability of the findings. TB IC practices in the study are self-reported based on the anonymous questionnaires rather than observation. Nevertheless, this is a first attempt to study HCWs’ KAP regarding TB IC in Nepal. The findings can serve as a baseline to design effective interventions to address the challenging issue of IC in relation to the possible threat of drug resistance and TB/HIV coinfection in the country. Further study at large scale preferably relying on observation of the IC practices is recommended.

## Conclusions

Overall knowledge and practices of HCWs on TB IC were not satisfactory with the knowledge level being even worse among non-medical and lower level staff. Regular skill-based training and orientation on TB IC for all cadres of HCWs can improve IC practices in the health facilities. In addition, TB IC policy or guideline is recommended to ensure effective IC measures in health facilities.

## Abbreviations

CHWs: Community health workers
DOTS: Directly observed treatment short-course
DR TB: Drug-resistant tuberculosis
HCW: Health care worker
HIV: Human immunodeficiency virus
IC: Infection control
KAP: Knowledge attitudes and practices
NTP: National Tuberculosis Program
SD: Standard deviation
SPSS: Statistical package for social sciences
TB: Tuberculosis
